# A Cohort Study of p53 Mutations and Protein Accumulation in Benign Breast Tissue and Subsequent Breast Cancer Risk

**DOI:** 10.1155/2011/970804

**Published:** 2011-08-22

**Authors:** Geoffrey C. Kabat, Rita A. Kandel, Andrew G. Glass, Joan G. Jones, Neal Olson, Catherine Duggan, Mindy Ginsberg, Abdissa Negassa, Thomas E. Rohan

**Affiliations:** ^1^Department of Epidemiology and Population Health, Albert Einstein College of Medicine, Bronx, NY 10461, USA; ^2^Department of Pathology and Laboratory Medicine, The Mount Sinai Hospital, Toronto, ON, Canada M5G 1X5; ^3^Center for Health Research, Kaiser Permanente Northwest, Portland, OR 97227, USA; ^4^Department of Pathology and Laboratory Medicine, Weill-Cornell Medical Center, New York, NY 10065, USA; ^5^Public Health Sciences, Fred Hutchinson Cancer Research Center, Seattle, WA 98104, USA

## Abstract

Mutations in the p53 tumor suppressor gene and accumulation of its protein in breast tissue are thought to play a role in breast carcinogenesis. However, few studies have prospectively investigated the association of p53 immunopositivity and/or p53 alterations in women with benign breast disease in relation to the subsequent risk of invasive breast cancer. We carried out a case-control study nested within a large cohort of women biopsied for benign breast disease in order to address this question. After exclusions, 491 breast cancer cases and 471 controls were available for analysis. Unconditional logistic regression was used to estimate odds ratios (OR) and 95% confidence intervals (95% CI). Neither p53 immunopositivity nor genetic alterations in p53 (either missense mutations or polymorphisms) was associated with altered risk of subsequent breast cancer. However, the combination of both p53 immunopositivity and any p53 nucleotide change was associated with an approximate 5-fold nonsignificant increase in risk (adjusted OR 4.79, 95% CI 0.28–82.31) but the confidence intervals were extremely wide. Our findings raise the possibility that the combination of p53 protein accumulation and the presence of genetic alterations may identify a group at increased risk of breast cancer.

## 1. Introduction

Mounting evidence suggests that cancer results from a series of mutations in genes involved in cell growth and differentiation, DNA repair, and cell cycle control [1, 2]. In the case of breast cancer, a series of genetic changes that develop over time is believed, in most cases, to drive the morphologic progression from proliferative disease without atypia to atypical ductal hyperplasia and then to ductal carcinoma in situ and ultimately invasive ductal carcinoma [3]. The actual sequence of genetic and molecular changes underlying the progression from normal breast tissue to invasive cancer has not been characterized, but the p53 tumor suppressor gene is thought to play a role [4].

Mutations in the p53 gene are among the most common genetic alterations found in breast cancer, occurring in 30–50% of cases of sporadic breast cancer [4, 5]. There is a correlation between the presence of p53 mutations and high histologic grade, lack of ER and/or PR expression, and less favorable prognosis [4, 6–11]. p53 mutations have varying effects, including prolonged expression of an altered p53 protein or, alternatively, the loss of protein expression [4]. Hence, p53 mutations do not necessarily result in p53 protein accumulation [5, 12–14], indeed, p53 protein accumulation has been found in association with missense but not truncation mutations [5, 14]. Although p53 mutations can occur at different locations in the p53 gene, most mutations tend to occur in the DNA-binding motifs within exons 5–8 [4, 6–11]. p53 mutations and/or p53 protein accumulation have been reported in 13% to 70% of invasive intraductal carcinomas of the breast [5, 15–23] and have also been detected in ductal carcinoma in situ [5, 20–22], in benign breast disease [24–28], in normal-appearing breast tissue [28], and in women at high risk of breast cancer [29]. Taken together, these findings suggest that p53 changes may play a role in the pathogenesis of breast cancer. 

Most previous studies of p53 in benign breast tissue have examined the prevalence of p53 immunopositivity and/or p53 mutations in case series, often involving only a limited number of cases [24–29]. Few studies have prospectively investigated the association of p53 immunopositivity and/or p53 alterations in women with benign breast disease in relation to the subsequent risk of invasive breast cancer. In two previous studies, we showed that women who were immunopositive for p53 in normal or benign breast tissue had a 2- to 2.5-fold increased risk of developing subsequent invasive breast cancer [30, 31]. p53 nucleotide changes overall were not associated with risk [31], whereas nonpolymorphic intronic changes in p53 were associated with increased risk of progression to invasive breast cancer [31]. Results of our previous analysis suggested that the combination of immunopositivity and mutation status identified a subgroup at increased risk of subsequent breast cancer better than either variable alone [31]. However, these results were based on a relatively small number (*n* = 104) of breast cancer cases. In the present study, conducted in a different study population comprising a large cohort of women biopsied for benign breast disease, we investigated the association between p53 protein accumulation and p53 mutations in exons 5 to 10 and subsequent risk of breast cancer.

## 2. Materials and Methods

### 2.1. Study Population

 We carried out a case-control study nested within a cohort of 15,809 women who received a breast biopsy either at Guy's Hospital (London, UK) or within the Kaiser Permanente Northwest (KPNW) health care system (Portland, OR USA). At Guy's Hospital, women were enrolled from 1946 to 1984 and at KPNW from 1970–1994. Women were eligible to participate if they had a histopathologic diagnosis of benign breast disease (BBD) on their index biopsy and were at least 21 years old at the time of the index biopsy. A detailed description of the study has been published previously [32].

### 2.2. Risk Factor Data

 Information on sociodemographic factors, reproductive and menstrual history, medical history, family history of breast cancer, and exogenous hormone use was obtained from medical records [33].

### 2.3. Followup and Ascertainment of Breast Cancer

 For the London cohort, the National Health Service Central Register provided information on breast cancer diagnoses and deaths of cohort members. In Portland, cases were ascertained through linkage to the KPNW cancer registry. Followup in London continued until 12/31/2003 and in Portland until 12/31/2001.

### 2.4. Case and Control Definition

Cases were women who had a biopsy for BBD with a subsequent diagnosis of in situ or invasive breast cancer. In both cohorts, controls were women who had a biopsy for BBD and who were alive but had not developed breast cancer during the same follow-up period as that for their corresponding case and they were individually matched to cases (1 : 1) on age and on age at diagnosis of BBD (with additional matching in the Portland cohort on the duration of membership in Kaiser Permanente health plan). The controls were selected with replacement and were eligible to be selected again as controls or to become cases subsequently. For this reason, although 1,065 women were selected from the two cohorts, there were a total of 1,092 records (Table 1). After exclusion of women who had breast cancer prior to baseline, who had no breast tissue, or were missing pathology data, 962 records were available for analysis (491 case records and 471 control records). Compared to women retained in the analysis, women who were excluded had a lower mean age at enrollment (39.0 versus 49.0 years), were more likely to be premenopausal (68.8 versus 52.1%), to be nulliparous (40.0 versus 19.5%), and to have had an earlier age at menarche 28.4 versus 19.7%, and were less likely to have a family history of breast cancer in a first-degree relative (5.9 versus 15%). 

### 2.5. Acquisition of Tissue and Histopathologic Review

For the present study, formalin-fixed paraffin-embedded blocks of benign breast tissue were retrieved from tissue archives. Hematoxylin and eosin-stained sections from the tissue blocks were reviewed histologically and classified according to the criteria developed by Page and Anderson [34] and without knowledge of the case-control status of the study subjects. Histology was categorized as nonproliferative/normal pathology, proliferative disease without atypia, and proliferative disease with atypia [32].

### 2.6. p53 Immunostaining

Sections (5 *μ*m) were cut from the paraffin blocks, mounted on aminopropyltriethoxysilane coated slides (2%; Sigma Chemical Co., St. Louis, Mo, USA), and deparaffinized. The sections underwent antigen retrieval (microwaved in 10 mmol/L citrate buffer (pH 6.0) for 15 minutes at a medium-high setting), and immunostaining was performed as described previously using antibody reactive with p53 (DO-7; monoclonal; dilution, 1 : 100; Novocastra Laboratories, Newcastle upon Tyne, UK) [30]. Immunoreactivity was detected using ABC Elite System (Vector, Calif) and DAB as the chromogen. Positive controls were sections from a paraffin-embedded breast cancer that was known to have a p53 mutation associated with p53 protein accumulation. Negative controls consisted of replacing the primary antibody with Universal Negative Control Mouse (Dako, Calif). Immunostaining and review of the immunostained slides were done without knowledge of the case-control status of the study subjects. Cytoplasmic staining was considered nonspecific and interpreted as negative. Any nuclear staining of epithelial cells was considered a positive reaction. The percentage of immunopositive cells was estimated and categorized into 0, >0–<1%, 1–9%, ≥10% total epithelial cells. p53 staining was present either in a few cells or all the cells of either a single or multiple ducts. Often the staining was seen in a single focus of ducts or in multiple foci within the tissue. We have reported previously that there was approximately 93% agreement (*κ* = 0.64) on the presence or absence of p53 immunostaining in the benign specimens reread by the same reviewer without knowledge of the result of the first reading [30].

### 2.7. p53 Mutation Analysis

Two 10 *μ*m-thick sections were cut from the paraffin blocks, dewaxed, and stained briefly in hematoxylin. The breast epithelium was microdissected from the stroma (for fibroadenomas, the entire lesion was taken), collected in a microfuge tube, and digested with proteinase K (Life Technologies, Burlington, ON, Canada; 0.5 mg/mL in 50 mmol/L Tris-HCl (pH 8.5), 10 mmol/L EDTA, 0.5% Tween 20) for at least 24 hours at 55°C. Proteinase K was inactivated by heating to 95°C for 15 minutes. The DNA was amplified by PCR using the primers for exons 5 to 10 and conditions listed in Table 2. The product was run on a 2% agarose gel, the band excised, and DNA extracted using Qiagen gel extraction kit (Qiagen, Miss, CAN). The purified DNA was manually sequenced using the CEQ DICS Quickstart kit (Beckman Coulter, CAN) according to the manufacturer's instructions in both directions using either sense or antisense primers and the CEQ8000 gene analysis system. If a change was detected, the DNA underwent repeat PCR and sequencing in the direction that had given the clearest profile in the first sequencing reaction. All sequences with a nucleotide change and approximately 20% of random samples negative for p53 alterations were rereviewed by another observer (RAK or SL). p53 mutation analyses were performed without knowledge of case-control status.

### 2.8. Subjects Available for Analysis

Of the 937 women (962 records) available for analysis, blocks of paraffin-embedded benign breast tissue were obtained and immunohistochemical/molecular analysis was completed for 884 women: 453 (92.3%) of the 491 cases and 431 (91.5%) of the 471 controls. Seventy-eight women (38 cases and 40 controls) were missing immunohistochemical results either because tissue samples were not obtained or because there was no breast epithelium in the tissue sections, rendering them unsuitable for immunohistochemical analysis. p53 sequencing results were available for 780 records (404 cases/376 controls) for exon 5, 788 records (399 cases/389 controls) for exon 6, 792 records (403 cases/389 controls for exon 7, 786 records (402 cases/382 controls) for exon 8, 797 records (407 cases/390 controls) for exon 9, and 719 records (366 cases/353 controls) for exon 10. Sequencing results for all exons were available for 651 records (329 cases/322 controls).

### 2.9. Statistical Analysis

In order to make use of all available data we carried out an unmatched analysis and estimated odds ratios (OR) and 95% confidence intervals (95% CI) for the association between immunohistochemically detected p53 protein accumulation and p53 changes detected by sequencing using unconditional logistic regression models [35]. p53 changes were examined overall (i.e., present/absent) and according to whether they were mutations or polymorphisms. The combined effects of immunopositivity and nucleotide alterations were also examined. Two sets of OR estimates were computed: (1) using models including terms for the matching factors (center, age at enrollment, and years of followup) and (2) using models including the matching factors, as well as histology (nonproliferative, proliferative), history of breast cancer in a first-degree relative, age at menarche, age at first live birth, number of pregnancies, and menopausal status (premenopausal, perimenopausal, and postmenopausal). (Women who reported having had a menstrual period within the last year, or who had had a hysterectomy without bilateral oophorectomy and were <45 years old, were classified as premenopausal. Women were classified as postmenopausal if they had ceased having menstrual periods at least 12 months earlier without surgical intervention, had had a bilateral oophorectomy, or had had a hysterectomy only and were >55 years old. The remaining women were classified as perimenopausal.) Matched analyses were also carried out using conditional logistic regression. Finally, we performed a sensitivity analysis excluding breast cancer cases diagnosed during the first 3 years of followup. All statistical tests were two-sided, and *P* values <0.05 were considered to be statistically significant. The design of the original study, laboratory assays, and statistical analysis all conform to the REMARK guidelines [36].

## 3. Results

Compared to controls, cases were more likely to have proliferative disease with atypia and to have a family history of breast cancer in a first-degree relative (Table 3). Cases were also more likely to have had a later age at first birth and to be nulliparous. Other factors differed little between cases and controls.

p53 immunopositivity was not associated with altered risk of subsequent breast cancer (Table 4). Furthermore, risk varied little by the percent of cells showing immunopositivity (Table 4). 

Presence of p53 staining (percent cells staining positive: 0, >0–<1, 1–9, ≥10) was strongly associated with presence of hyperplasia (present versus absent, *P* < 0.0001). The proportions of women with hyperplasia (present/absent) by percent cell staining positive were 57.8%/86.1%, 21.1%/6.5%, 14.9%/5.9%, and 6.2%/1.5%. Overall histological category (nonproliferative, proliferative without atypia, atypical hyperplasia) was also associated with the presence of p53 staining, *P* = 0.03. Presence versus absence of any genetic alteration was not associated with histological category—*P* = 0.88 (data not shown). 

For cases and controls, the proportions showing any p53 alteration on mutation analysis were 4.8% and 5.5%, respectively (Table 5). Of the alterations in cases, 17 were missense mutations and 7 were polymorphisms; the corresponding numbers for controls were 16 and 7. Risk of breast cancer in association with these alterations did not differ from the null. No deletions, insertions, nonsense mutations, silent mutations, or splice site mutations were detected. 

When p53 protein accumulation and nucleotide changes were examined jointly, compared to women who had neither p53 immunopositivity nor any p53 nucleotide change, women who were positive for one variable but not the other were not at increased risk of subsequent breast cancer (Table 6). Only 3 cases and 2 controls had evidence of both immunopositivity and a nucleotide change. The combination of both p53 immunopositivity and any p53 nucleotide change was associated with an approximate 5-fold, nonsignificant increase in risk (fully adjusted OR 4.79, 95% CI 0.28–82.31) with extremely wide confidence intervals. 

Results of the matched pair analyses were generally consistent (both in direction and magnitude of the risk estimates) with those of the unmatched analyses. 

When cases diagnosed within the first 3 years of followup were excluded from the analysis, the results were unchanged (data not shown).

## 4. Discussion

In this large case-control study nested within a cohort of women biopsied for benign breast disease, p53 immunopositivity was not independently associated with increased risk of breast cancer. Mutation analysis revealed a low frequency of p53 changes, and these were not associated with increased risk. However, there was a suggestion that the combination of immunopositivity and the presence of a p53 mutation was associated with substantially increased risk, but the number of women in this group was small and the confidence intervals were very wide. 

 In an earlier case-control study nested within a cohort of 4,888 women diagnosed with benign breast disease [30], p53 protein accumulation was associated with an increased risk of progression to breast cancer (OR 2.55, 95% CI 1.01–6.40). Similar changes were not observed in the current study likely because of the differences in the exons evaluated and the primers utilized. In the original study exon 4 was also evaluated and the primers utilized also included the flanking intronic regions. In a subsequent analysis of the same cohort [31], p53 immunopositivity in benign breast tissue was associated with a 2-fold increase in the risk of subsequent breast cancer, whereas the presence of any type of p53 nucleotide change in the benign tissue was not associated with altered risk. When immunoreactivity and nucleotide changes were examined jointly, however, those with both p53 immunopositivity and a p53 nucleotide change (all changes combined) had a 3-fold increase in breast cancer risk [31]. We are aware of three other prospective studies of p53 changes or protein accumulation in normal or benign breast tissue in relation to risk of subsequent breast cancer [25, 27, 29], but these had had small numbers of breast cancer cases.

The most common p53 mutations occurring in sporadic breast cancer cases are missense point mutations or inframe deletion/insertion mutations, which account for 53–73% of all p53 mutations in these subjects [4]. Because the p53 proteins expressed by these mutant genes have a prolonged half life, they are detectable using immunohistochemical techniques. However, 27–47% of p53 mutations involve nonsense point mutations or frameshift deletion/insertion mutations that result in truncated proteins, which are not detected by immunohistochemistry [4]. In view of the limited concordance between the presence of mutations and the presence of protein overexpression and the many points at which p53 function can be disrupted, the joint effect of both factors may better identify a group at increased risk of invasive breast cancer. Findings from our earlier analysis [31] as well as our current results appear to support this. However, the small number of women exhibiting both immunopositivity and any genetic alteration in both studies precluded the examination of specific types of mutations.

Most benign breast disease is associated with only a moderately increased risk of progressing to cancer, whereas the small subgroup with atypical hyperplasia is at substantially increased risk of progression [27, 32]. We previously reported that, relative to women with BBD/normal pathology, women with proliferative lesions but without atypia had a multivariable-adjusted odds ratio for breast cancer of 1.45 (95% CI 1.10–1.90) and women with atypical hyperplasia had an odds ratio of 5.27 (95% CI 2.29–12.15) [32]. In spite of the large size of the cohort from which cases and controls were selected for the present study, the number of women with atypical hyperplasia was too small to permit separate analysis of this subgroup. For this reason, extended followup of large cohorts or pooling of a number of cohorts of women with benign breast disease is needed to make it possible to examine p53 protein accumulation and mutations, and their combined effects, in the subset of BBD with the highest risk of progression (i.e., those with atypical hyperplasia).

It has been proposed that mutations in the p53 gene observed in benign breast tissue may lead to genetic instability and defective DNA repair and, ultimately to clonal expansion of transformed cells and progression to invasive breast cancer [24]. Point mutations can contribute to the inactivation of tumor suppressor gene function or to the activation of protooncogenes [4], whereas DNA amplification can activate protooncogenes [4]. However, Soussi [37, 38] has recently argued that the role of p53 genetic alterations in carcinogenesis is much more complicated than has been generally recognized. He emphasized that there is a broad spectrum of heterogeneous p53 mutations and that the effects of these mutations can be modified by gene-gene interactions, protein-protein interactions, and tissue-specific factors. This suggests that a more comprehensive assessment and understanding of the role of p53 alterations in the etiology of breast cancer would require simultaneous assessment of the complex network of overlapping pathways and regulating factors.

Strengths of the current study include the large cohort of women who underwent a breast biopsy at two collaborating centers and were followed for an average of 14 years, the availability of immunohistochemical and p53 mutation results for >90% of cases and controls, and the ability to adjust for major breast cancer risk factors. Adjustment for these risk factors appeared to attenuate the OR for missense mutations. An additional strength is the fact that p53 analyses were performed without knowledge of case-control status, making differential bias in the assessment of p53 status unlikely.

Sequencing was used to identify gene alterations, but this method is influenced by the amount and quality of DNA. Furthermore, the number of samples suitable for analysis was limited by the age of the samples and by variation in the types of fixatives. Ninety-nine percent of samples from Portland were formalin fixed, and 99% of those from London were fixed with Formal saline. We repeated the analysis of immunopositivity (present/absent) and of any genetic alteration (present/absent) by center, and the results did not differ from the overall results, although the statistical power was limited.

Several additional limitations of the study should be mentioned. Paraffin-embedded blocks of benign breast tissue were not obtained for 8% of cases and controls. When women lacking immunohistochemistry results (*N* = 78) were compared to those with results (*N* = 884) with respect to baseline characteristics, apart from a difference in mean age (55.7 for the former and 48.4 for the latter), few differences were seen. When women lacking sequencing results (*N* = 318) were compared to those with sequencing results (*N* = 644), again, aside from a difference in mean age (52.1 for the former versus 47.4 for the latter), other background characteristics were similar. Finally, the small number of women who showed both immunopositivity and genetic alterations precluded firm conclusions about the effect of these factors in combination and, also, precluded examination of specific mutations.

In conclusion, the findings from this study suggest that the combined assessment of p53 overexpression and mutations in women with normal or benign breast tissue may identify a subgroup at increased risk of developing invasive breast cancer. However, the functional significance of different genetic alterations and their possible role in the progression culminating in invasive breast cancer needs to be elucidated. It also remains to be determined whether p53 immunohistochemical and mutation analysis can improve on the use of histology in the clinical assessment of a woman's risk of breast cancer.

## Figures and Tables

**Table 1:** Number of cases and controls by center and exclusions.

	London	Portland	Total
*Number of unique individuals*	452	613	1065
(i) Number of controls used an additional 1×	0	7	
(ii) Number of controls used an additional 2×	0	3	
(iii) Number of controls that reappear as cases	8	6	

*Total number of records*	460	632	1092
(i) Number of case records	230	316	546
(ii) Number of control records	230	316	546

*Exclusions* (breast cancer before baseline, no breast tissue, missing pathology data)	128	2	130
(i) Cases records excluded	53	2	55
(ii) Control records excluded	75	0	75

*Total number of records after exclusions (used in unmatched analysis)*	**332**	**630**	**962**
(i) Cases after exclusions	177	314	491
(ii) Controls after exclusions	155	316	471
*Total number of women after exclusions*	**326**	**611**	**937**
*Total number of case-control sets (used in matched analysis)*	148^1^	316^1^	464

^1^Matched pairs only.

**Table 2:** p53 primer sequences and PCR conditions.

Primers	Sequences	Size	PCR conditions
Exon 5	5′-GCTGCCGTGTTCCAGTTGCT-3′	294 bp	95°C, 50 sec; 58°C, 50 sec; 72°C, 60 sec, 35 cycles
	5′-CCAGCCCTGTCGTCTCTCCA-3′		

Exon 6	5′-GGCCTCTGATTCCTCACTGA-3′	199 bp	95°C, 50 sec; 55°C, 50 sec; 72°C, 60 sec, 35 cycles
	5′-GCCACTGACAACCACCCTTA-3′		

Exon 7	5′-TGCCACAGGTCTCCCCAAGG-3′	196 bp	95°C, 50 sec; 56°C, 50 sec; 72°C, 60 sec, 35 cycles
	5′-AGTGTGCAGGGTGGCAAGTG-3′		

Exon 8	5′-CCTTACTGCCTCTTGCTTCT-3′	225 bp	95°C, 50 sec; 55°C, 50 sec; 72°C, 60 sec, 35 cycles
	5′-ATAACTGCACCCTTGGTCTC-3′		

Exon 9	5′-GCCTCAGATTCACTTTTATCACC-3′	152 bp	95°C, 50 sec; 56°C, 50 sec; 72°C, 60 sec, 35 cycles
	5′-CTTTCCACTTGATAAGAGGTCCC-3′		

Exon 10	5′-TGATCCGTCATAAAGTCAAACAA-3′	236 bp	95°C, 50 sec; 56°C, 50 sec; 72°C, 60 sec, 35 cycles
	5′-GGAGTAGGGCCAGTAAGGG-3′		

**Table 3:** Baseline characteristics of the study subjects.

Variable	Cases (*N* = 491)		Controls (*N* = 471)		*P* value*
	*N*	(%)	*N*	(%)	
Age at biopsy					
<40	109	(22.2)	105	(22.3)	
40 to <50	169	(34.4)	166	(35.2)	
50 to <60	107	(21.8)	102	(21.7)	
60 to <70	69	(14.1)	62	(13.2)	
≥70	37	(7.5)	36	(7.6)	0.99
Histology					
Nonproliferative disease	137	(27.9)	173	(36.7)	
Proliferative disease					
without atypia	322	(65.6)	289	(61.4)	
Proliferative disease					
with atypia	32	(6.5)	9	(1.9)	<0.0001
Age at menarche					
<12	76	(20.0)	74	(19.5)	
12	87	(22.9)	77	(20.3)	
13	109	(28.7)	116	(30.5)	
≥14	108	(28.4)	113	(29.7)	0.80
Missing	111		91		
Age at first live birth					
Nulliparous	95	(21.9)	73	(17.3)	
≥30	58	(13.4)	34	(8.1)	
25–<30	88	(20.3)	96	(22.8)	
20–<25	150	(34.6)	177	(41.9)	
<20	43	(9.9)	42	(10.0)	0.02
Missing	57		49		
Parity					
Nulliparous	95	(19.3)	73	(15.4)	
Parous	392	(79.8)	391	(83.0)	0.13
Missing	4		7		
Number of pregnancies					
None	95	(19.8)	73	(16.3)	
1	80	(16.4)	69	(14.8)	
2	152	(31.1)	157	(33.6)	
3	83	(17.0)	91	(19.5)	
≥4	77	(15.7)	74	(15.8)	0.83
Missing	4		7		
Menopausal status					
Premenopausal	264	(53.8)	237	(57.0)	
Perimenopausal	39	(7.9)	34	(7.2)	
Postmenopausal	188	(38.3)	200	(38.3)	0.27
Ever used hormone therapy					
No	350	(71.3)	317	(67.3)	
Yes	141	(28.7)	154	(32.7)	0.18
Family history of breast cancer in a first-degree relative					
No	386	(82.7)	394	(83.8)	
Yes	81	(17.3)	57	(12.0)	0.05
Missing	24		20		

*Based on likelihood-ratio-chi-square test.

**Table 4:** p53 immunopositivity in benign breast tissue and risk of breast cancer.

Variable	Level	No. of cases	No. of controls	OR^a^ (95% CI)	OR^b^ (95% CI)
Immunopositive	Absent	370	345	1.0 (reference)	1.0 (reference)
	Present	83	86	0.95 (0.62–1.44)	0.88 (0.57–1.37)
	Missing	38	40		
% cells immunopositive					
	0	370	345	1.00 (reference)	1.00 (reference)
	>0–<1	42	39	1.25 (0.69–2.26)	1.27 (0.68–2.35)
	1–9	30	37	0.75 (0.40–1.39)	0.68 (0.36–1.32)
	≥10	11	10	0.74 (0.26–2.10)	0.57 (0.20–1.64)
	Missing	38	40		

^a^Adjusted for center, age at enrollment, and years of followup.

^b^Adjusted for age at enrollment, center (city), years of followup, histology (nonproliferative, proliferative disease), family history (no, yes), age at menarche (<12,12,13,14–19), age at first birth (continuous), number of pregnancies (continuous), and menopausal status (premenopausal, perimenopausal, postmenopausal).

**Table 5:** Association of p53 exonic changes in DNA extracted from benign breast tissue and breast cancer.

Type of exonic change	Level	No. of cases	No. of controls	OR^a^ (95% CI)	OR^b^ (95% CI)
Any change	Wild type	301	294	1.0	1.0
	Present	24	25	1.46 (0.60–3.56)	1.03 (0.40–2.65)
	Missing	166	152		

Deletions	Wild type	301	294	NA	NA
	Present	0	0		
	Missing	190	177		

Insertions	Wild type	301	294	NA	NA
	Present	0	0		
	Missing	190	177		

Nonsense mutations	Wild type	301	294	NA	NA
	Present	0	1		
	Missing	190	176		

Missense mutations	Wild type	301	294	1.0	1.0
	Present	17	15	2.42 (0.89–7.29)	1.47 (0.48–4.52)
	Missing	173	162		

Silent mutations	Wild type	301	294	NA	NA
	Present	0	1		
	Missing	190	176		
					

Splice site mutations	Wild type	301	294	NA	NA
	Present	0	0		
	Missing	190	177		

Polymorphism	Wild type	301	294	1.0	1.0
	Present	7	7	0.59 (0.12–2.86)	0.47 (0.08–2.96)
	Missing	183	170		

NA: not applicable.

^a^Adjusted for center, age at enrollment, and years of followup.

^b^Adjusted for age at enrollment, center (city), years of followup, histology (nonproliferative, proliferative disease), family history (no, yes), age at menarche (<12,12,13,14–19), age at first birth (continuous), number of pregnancies (continuous), and menopausal status (premenopausal, perimenopausal, postmenopausal).

**Table 6:** Association of p53 protein accumulation and p53 nucleotide changes in benign breast tissue and risk of breast cancer.

p53 change	Level	No. of cases*	No. of controls*	OR^a^ (95% CI)	OR^b^ (95% CI)
Immunopositivity and/or nucleotide change	Both absent	235	227	1.0 (reference)	1.0 (reference)
	Immuno +, change −	65	63	1.17 (0.73–1.91)	1.10 (0.66–1.84)
	Immuno −, change +	21	23	1.35 (0.53–3.44)	0.89 (0.33–2.40)
	Immuno +, change +	3	2	5.60 (0.27–115.3)	4.79 (0.28–82.31)
	Missing	167	156		

*38 cases/40 controls missing information on immunopositivity; 0 cases/0 controls missing information on nucleotide changes; 38 cases/40 controls missing information on combined variable.

^a^Adjusted for center, age at enrollment, and years of followup.

^b^Adjusted for age at enrollment, center (city), years of followup, histology (nonproliferative, proliferative disease), family history (no, yes), age at menarche (<12,12,13,14–19), age at first birth (continuous), number of pregnancies (continuous), and menopausal status (premenopausal, perimenopausal, postmenopausal).
